# The Health Communications Assessment Checklist: a teaching tool for uncovering bias in the age of AI

**DOI:** 10.3389/fpubh.2026.1872506

**Published:** 2026-08-27

**Authors:** Rebecca H. Ofrane, Taliah Canada, Amanda S. Birnbaum

**Affiliations:** Department of Public Health, College for Community Health, Montclair State University, Montclair, NJ, United States

**Keywords:** assessment, diversity as an asset, health communication, teaching, use of technology innovation

## Abstract

**Introduction:**

The COVID-19 pandemic highlighted significant failures in United States public health communications, which were often perceived as unclear and biased, particularly among racialized and marginalized groups. As generative artificial intelligence (genAI) becomes integrated into public health practice, there is an urgent need to train the workforce to recognize and mitigate the societal biases these tools can reproduce. This study describes the development and classroom testing of a tool designed to teach these critical evaluation skills.

**Methods:**

We developed the “Health Communications Assessment Checklist,” a 14-question pedagogical tool based on the CommunicateHealth Framework for Equity-Centered Health Communication and the CDC’s Guiding Principles for health equity. The checklist was tested during the 2024–2025 academic year in two graduate-level courses (*n* = 55 students) at a large public university. The single-lesson exercise required students to use ChatGPT-4o to generate health communication content and then apply the checklist to critique the output for clarity, relatability, actionability, and inclusivity.

**Results:**

Post-exercise surveys and classroom discussions indicated positive student engagement and self-reported skill-building in critical assessment. A key finding was that students leaned heavily on their diverse individual identities and lived experiences to identify subtle biases that the AI generated. Furthermore, the checklist acted as a self-assessment tool, helping students identify gaps in their own knowledge of communication implementation. Feedback led to iterative improvements of the tool.

**Conclusion:**

The Health Communications Assessment Checklist is a simple, modifiable, and promising pedagogical tool for public health education. By centering equity and reflexivity, it helps prepare the future workforce to critically manage AI-generated content, ensuring health communications are inclusive and effective in promoting health equity.

## 1 Introduction

As the field of public health reflects back on the COVID-19 pandemic and our local and national response, we are learning from both our successes and failures. From the perspective of health promotion, one of the primary lessons is the importance of health communications (1–3).

The public health response at the time is now viewed as having been unclear, biased, and arbitrary, and is seen as a major failure of the United States public health system (4–6). Racialized and marginalized groups bore the greatest burden of COVID-19, replicating and sometimes widening the health disparities that were already evident pre-pandemic (7, 8). As we seek to restore faith and trust in public health, it is critical to proactively recognize and remove bias in health communications (9, 10).

At the same time, we see the rise of generative artificial intelligence (genAI) programs integrating into educational and workplace environments, for purposes including communications and content generation for public health (11, 12). For the current generation of public health graduate students, it is now important to obtain skills in critically evaluating health communications - including genAI output - for bias as well as accuracy (13). These skills are necessary for public health professionals to communicate clearly, rebuild trust with the public, and ensure equitable understanding of scientific data and information.

While genAI programs like ChatGPT are capable of producing vast results at rapid speeds, they lack key critical thinking mechanisms like human understanding and ethical reasoning. Outputs are generated based on patterns in training data, i.e., online content, meaning societal biases in the data are reproduced in the output, and without a critical human eye, these biases are recycled in the AI-generated content (14). For example, generative AI systems may reproduce stereotypes or omit contextual information, compromising both fairness and reliability. For this reason, it is important that students are able to evaluate the information they receive from genAI.

The same can be said for health communications more broadly. In a time where health information is widely accessible through media, public campaigns, and digital platforms, it is necessary to be able to recognize bias. Health communication messaging, which influences public perceptions, health behaviors, and even policy decisions, may not always be free from bias or error. Without careful critique of the health message in the context of the audience and delivery setting, health communications are susceptible to both subtle and overt bias. Unchecked bias in health communications can also contribute to widening health disparities (10, 15, 16). Messages that fail to consider cultural values and practices, language barriers, or socioeconomic constraints – as well as messages that are steeped in stereotypes – run the risk of excluding or alienating vulnerable populations. Critiquing these communications is therefore not just an academic exercise, but a necessary step toward more equitable and accurate health communication.

Within this context, we recognized an opportunity to incorporate a practical lesson for graduate students on using genAI to generate health communication content, iteratively critique the output for bias, and make active decisions about using generated content. We developed and tested a new Health Communications Assessment Checklist designed to support both students and public health professionals in efficiently evaluating health communications for equity and bias. This article describes the process of developing, testing and refining the checklist based on student feedback. Additionally, we provide guidance for instructors across diverse educational settings to implement the exercise, alongside practical recommendations for public health practitioners utilizing the tool in professional practice.

## 2 Pedagogical framework

**Figure 1:**
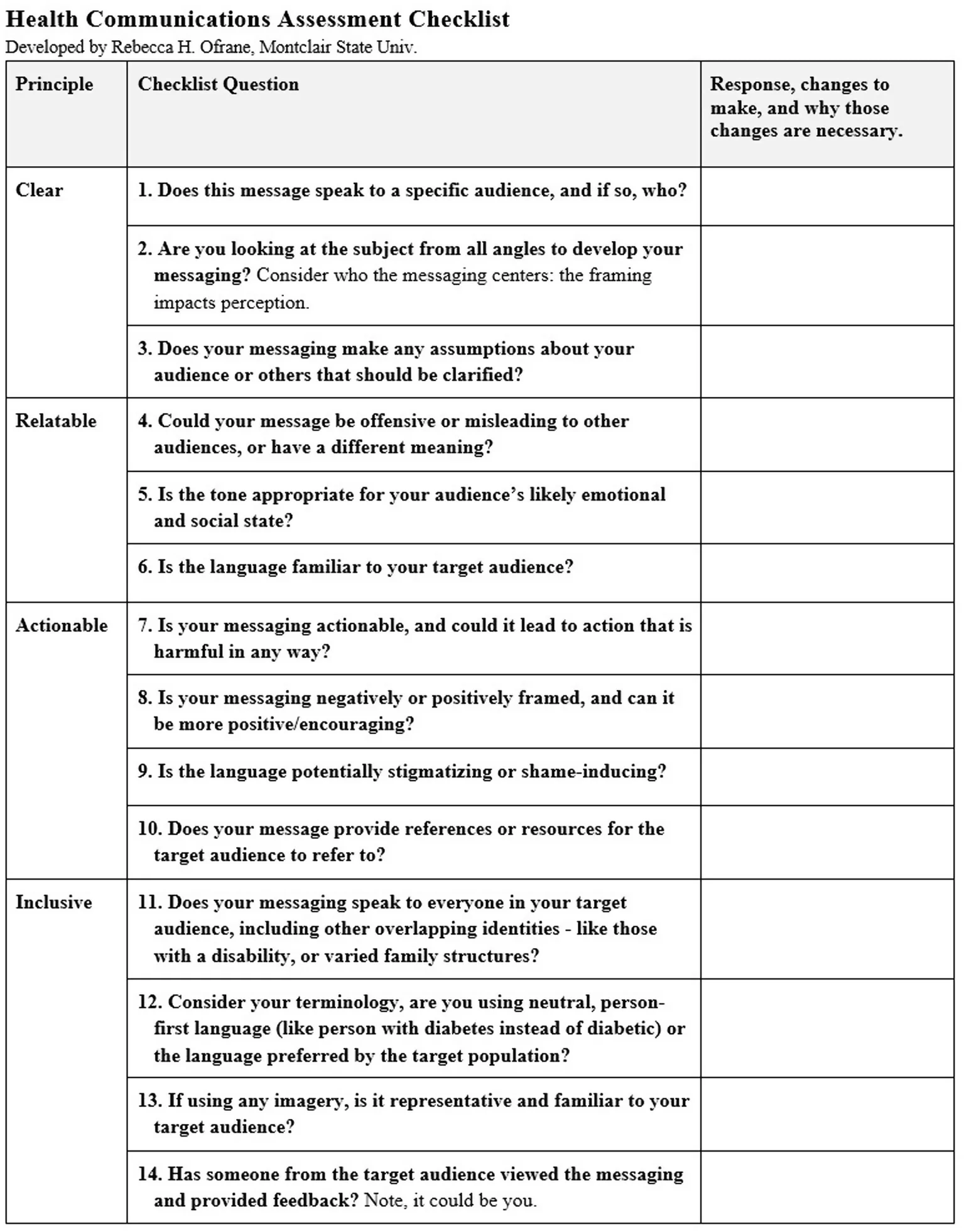
The Health Communications Assessment Checklist, developed by author RH Ofrane, as described through this paper.

The checklist was developed in the 2024–25 academic year based on a review of existing tools for teaching graduate students to recognize bias in health communications. Two primary documents came to light. First, CommunicateHealth’s Framework for Equity-Centered Health Communication describes the need for communications that are clear, relatable, actionable, and inclusive (17). The Framework provides a detailed process for the development of communication products that meet these criteria. Second, the CDC’s Guiding Principles to Promote an Equity-Centered Approach to Public Health Communication (18), a resource designed to help public health professionals consider social inequities and adapt to the cultural, linguistic, and historical context of each audience of focus. Together, these frameworks provide foundational understanding and language for health equity and human-centered communications design. They also make explicit the need for reflexivity on the part of the workforce members designing the communications. Based on these resources, we designed the Health Communications Assessment Checklist (“the checklist,” see Figure 1). The checklist is a simple tool with 14 critical and reflexive questions, focused on designing communications that are clear, relatable, actionable, and inclusive.

While this paper focuses on the development and refinement of the Checklist which can be used in different settings and by many types of users, we also present pedagogical strategies for teaching purposes. One pedagogical goal was to develop a single-lesson activity that could easily fit into a variety of courses. Rather than replacing activities already in place to teach and assess the foundational health communication competencies required for accreditation, we viewed this activity as an additional, cross-curricular opportunity for students to learn and practice health communications skills. Thus, we created the simplified, single-page checklist that we introduced and applied together in one class session. The learning activities constitute a classroom-based experiential learning process, using collective experience and reflection activities (20). For a more thorough process guide or background reading for students, the Framework for Equity-Centered Health Communication is an excellent publication that might better serve a full course on health communications and program planning.

## 3 Learning environment

We introduced and tested the checklist in two different graduate public health courses–Social and Behavioral Sciences in Health which typically enrolls first-year graduate students, and Culminating Seminar which enrolls students preparing to graduate. These were comprised of two sections of the first-year students (*n* = 40) which were conducted in person, and one section of the culminating seminar (*n* = 15), which was conducted online. Prior to the class sessions where the activity was implemented, students had a variety of experience with generating health communications. In Social and Behavioral Sciences in Health, students discuss communications as an important strategy to affect health behaviors within different social contexts and populations. In Culminating Seminar, students are applying and synthesizing competencies from their entire master’s program rather than being taught new skills. But nearly all students had some baseline instruction on health communications, conceptually.

**Figure 2:**
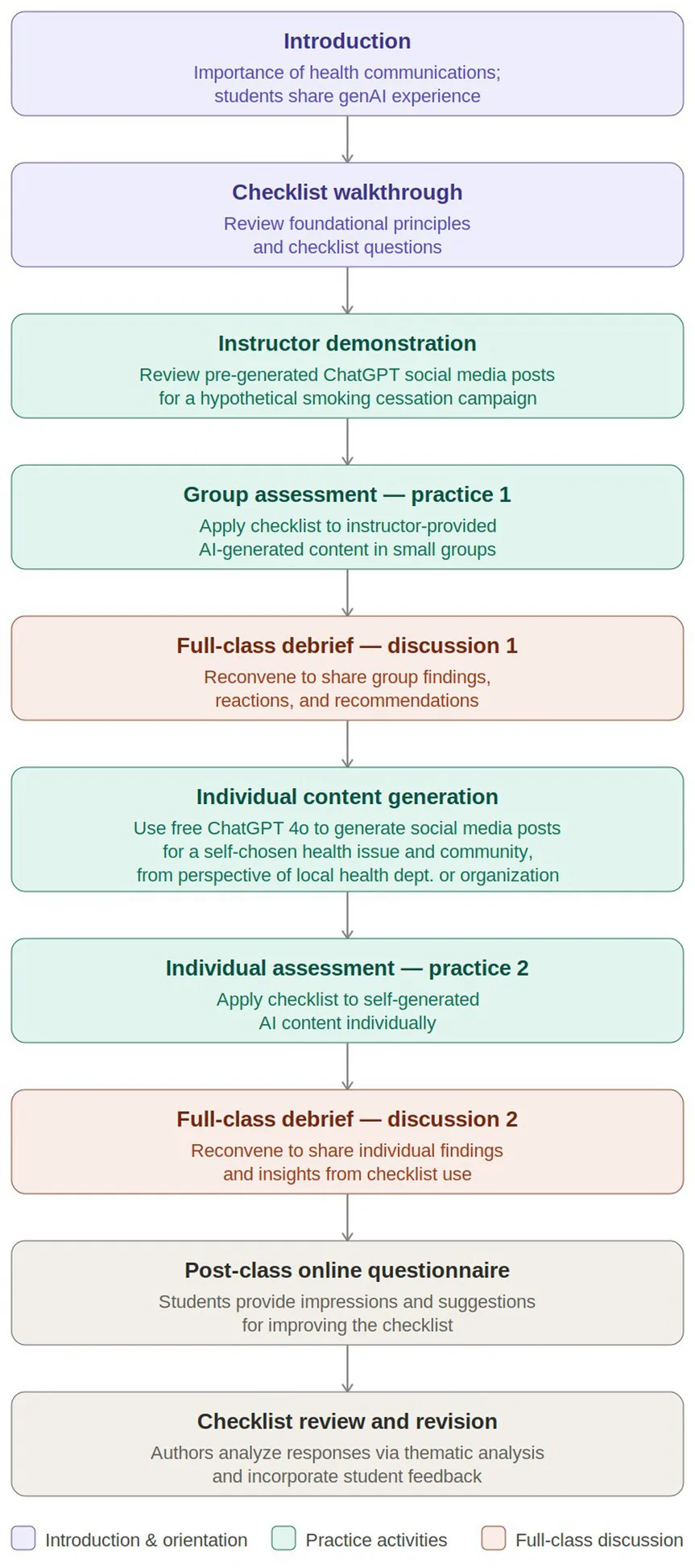
Instructional diagram showing the steps followed for teaching graduate students how to use the Health Communications Assessment Checklist for critical purposes.

For context, we are a large, public, Hispanic-Serving Institution, defined as enrolling over 25% Hispanic students. The same steps were used in each of the three classes. See Figure 2 for an instructional diagram.

To introduce the activity, instructors reviewed the importance of health communications and asked students to share their experiences using genAI. We then walked through the checklist and its foundational principles and questions. The instructor then showed students a series of social media posts for a hypothetical neighborhood smoking cessation campaign, which had been generated by ChatGPT in a work session prior to class. Students shared initial reactions, then formed groups and used the checklist to assess the genAI-produced social media content, noting their responses and recommendations to improve the communications.

Following the group work, we reconvened as a full class to debrief about students’ findings and insights. We then charged students with generating their own set of social media content, for a hypothetical campaign around a health issue and population or community of their choosing. They were specifically instructed to practice generating health communications from the perspective of a local health department or community organization. We instructed them to use the free version of ChatGPT 4o to generate this content, and then use the checklist to assess the new AI-generated content. We then reconvened for a second full-class discussion. This process gave students two opportunities to practice using the checklist, in both group and individual settings.

## 4 Methods

After the class sessions, students responded to an online questionnaire about the learning experience and the checklist (the survey was determined exempt by Montclair State University #IRB-FY24-25-3994). Qualitative responses were analyzed using Braun and Clarke (19) thematic analysis framework (2022) within Dedoose software. Two coders (authors RHO and ASB) independently reviewed a subset of responses to inductively generate initial codes, which were then consolidated into a structured codebook through iterative discussion. Using the finalized codebook, the coders independently analyzed the full dataset, resolving discrepancies through consensus meetings. Student suggestions for improving the checklist were reviewed and incorporated by the instructors, resulting in the final version presented in Figure 1.

## 5 Results and discussion

### 5.1 Results

There were 49 eligible student responses, with students representing a wide diversity of race/ethnicity, age, language and nativity. Demographic groupings include students of color (reporting any race or ethnicity besides white), primary language, age (differentiating between those born before or after 2000, to assess generational differences in technology usage), and United States- or foreign-born. 73.5% of the students in our sample identified as a person of color (*n* = 36), and 26.5% as white (*n* = 13); 71.4% of students’ primary language was English, 66% were born in the year 2000 or later, and 26.5% were foreign-born.

Reflecting this diversity, a key result of the class exercise and discussion was that many students explicitly drew upon their own individual identities as part of their process of determining bias. Students recognized the value of their own diverse identities through the exercise. As one student stated, “I strongly believe that our identity and culture are what makes us unique when critiquing AI output. An individual’s identity is personalized by their own lived experiences, implicit biases they have faced, the challenges they overcome, and how their cultural background has influenced their personal life as well as their societal, political, and emotional viewpoints.” There was awareness of their own biases, as well as appreciation for their own identities. Another student echoed this connection, “I’m a black woman and Haitian immigrant, so I connect a lot to those communities and I can just feel when messaging feels off or is not written with those audiences in mind.”

A second finding was how the use of an explicit checklist prompted students to assess their own skills and identify gaps in their knowledge of communications principles. Most of the suggestions and recommendations about improving the checklist focused not on whether or not bias was present, but rather how to implement some of the recommended actions. One student specifically suggested an ironic technical adaptation to the workflow: “I might modify the checklist by running the information back through AI and ask if it detected any bias. I’m not sure what would happen if you did so, but it would be interesting to see what it had to say!”

Additionally, a few students suggested adding an explicit prompt about accuracy and fact-checking to the checklist, noting that checking for factual correctness was a distinct step from evaluating equity and tone.

Lastly, student feedback confirmed successful skill-building and retention of the communication and equity concepts taught through the use of the checklist. Several students across classes expressed surprise at the output, both in terms of speed and bias. In particular, many stated concern about others who use gen-AI without first being taught critical assessment skills like those they learned in this class session. One student reflected, “I am also concerned about people who use this messaging without taking into account the checklist and adding to cultural misconceptions and stereotypes without even noticing.”

### 5.2 Discussion and recommendations

Based on student feedback, several practical strategies emerge for educators integrating AI evaluation tools into health communication or public health coursework. First, instructors could consider pacing the curriculum to separate conceptual instruction from practical application. Student feedback revealed that most questions focused on *how* to implement recommended actions rather than identifying bias itself, suggesting that students may not have initially recognized foundational communication concepts as framed in the checklist or lacked confidence modifying AI output to be relatable, actionable, and inclusive. Introducing the checklist and its core equity concepts in a discrete class session, prior to asking students to apply it, can build necessary foundational confidence.

Additionally, instructors can enhance critical engagement by incorporating AI self-auditing exercises into the classroom workflow. Building on student suggestions to re-prompt generative models, instructors might design an activity where students ask the AI to critique its own generated content for potential bias. Comparing student-identified biases with AI-identified biases serves as an engaging teaching moment regarding the limits of automated self-correction. Finally, educators should consider integrating fact-checking into equity evaluation frameworks. Although assessing equity and bias requires a distinct skillset from traditional fact-checking, the rise of targeted disinformation campaigns aimed at specific populations means factual accuracy is inherently bound to eliminating harmful communication bias. Because students are unlikely to use multiple evaluation tools simultaneously in real-world workflows, expanding the checklist to include an accuracy and fact-checking component—or dedicating a follow-up lesson to verifying AI-generated facts—ensures a more comprehensive evaluation process.

Beyond higher education, student feedback also offers valuable insights for public health practitioners using the checklist as a pre-release auditing tool. Student responses underscored the critical risks of disseminating unvetted AI output, with participants expressing strong concern regarding individuals using generative AI without critical assessment skills. Automated output can easily reinforce cultural misconceptions or adopt an improper tone if left unchecked. Practitioners using AI for campaign development can utilize the checklist’s four domains (Clear, Relatable, Actionable, Inclusive) to systematically catch these nuances, particularly by reviewing tone relative to an audience’s emotional state and screening for subtle, stigmatizing language. Furthermore, because student responses revealed that identifying bias is easier than knowing how to implement inclusive revisions (e.g., transitioning to neutral, person-first terminology or representing varied family structures), practice settings should treat the checklist not as a simple pass/fail gate, but as a framework for collaborative team review and skill-building. Finally, given student insights regarding the intersection of bias and targeted disinformation, practitioners must pair equity checks with rigorous fact-checking before public release to ensure messaging is not only culturally resonant, but factually sound.

Finally, while this evaluation was conducted within a Hispanic-Serving Institution (HSI) context, the core principles of the checklist are highly transferable across diverse educational and professional landscapes. The framework can be seamlessly adapted for undergraduate public health curricula to build introductory health literacy and communication skills, as well as integrated into continuing education or workforce training workshops for active public health professionals. In non-HSI or less demographic-diverse learning environments, the structured prompt questions serve as an essential scaffold, encouraging learners to step outside their lived experiences and systematically audit communications for populations whose perspectives may not be natively represented in the room.

### 5.3 Strengths and limitations

This study on the use of the Health Communications Assessment Checklist has strengths and limitations. First, it is a relevant and practical method of teaching critical AI literacy skills, particularly for students in a similar university setting. While AI literacy skills will continue to evolve quickly, the Checklist provides a tool that students can use with any AI model or iteration.

One limitation is our relatively small sample size in a single institution, which we aimed to address through assessing students on the same activity in different classroom settings and courses. Another consideration is our utilization of a single AI model–ChatGPT. This was an intentional choice, including both the single model and the use of the free version, so that we could compare output and student interaction with a single model. Our aim was not to analyze the nature of the bias in different AI model outputs, but instead to use the Checklist to surface and identify biases in the communication output more generally. Lastly, our analysis relies on self-reported student questionnaire responses and classroom discussions, rather than objective assessment of skill improvement.

Future implementation and evaluation of the Checklist could include additional evaluation measures like pre- and post-surveys, longer-term follow-up on student learning outcomes and retention, and integration into professional health communication practices.

## 6 Conclusion

The Health Communications Assessment Checklist is a simple, modifiable tool for teaching public health students (among other fields) and practitioners the knowledge and practical skill of communication assessment for bias. As generative AI tools become increasingly embedded in campaign development, the burgeoning public health workforce requires critical evaluation skills to ensure messaging is factually sound, non-stigmatizing, and culturally resonant across diverse populations. Recognizing the unique value of one’s own identity and perspective in this evaluation process serves as a key benefit of the exercise from a pedagogical lens. While tested within a graduate Hispanic-Serving Institution (HSI) context, the checklist framework is highly transferable across the educational and professional continuum. It offers an effective scaffold for undergraduate health literacy courses, non-HSI or less demographic-diverse learning environments, and continuing-education workshops for active public health professionals. The authors encourage instructors and practitioners alike to utilize the checklist and exercise as presented here, or to adapt it based on specific institutional settings, student needs, and evolving communication formats. By embedding this critical evaluation skillset into higher education and workforce practice, we aim to enhance both the equity and effectiveness of public health practice.

## Data Availability

The raw data supporting the conclusions of this article will be made available by the authors, without undue reservation.
